# Organ Donation Awareness: Rethinking Media Campaigns 

**DOI:** 10.15171/ijhpm.2018.85

**Published:** 2018-09-08

**Authors:** Emmanouil K. Symvoulakis, Adelais Markaki, Dimitrios Anyfantakis, George Rachiotis

**Affiliations:** ^1^Private Family Practice Unit in Heraklion, Heraklion, Crete, Greece.; ^2^School of Nursing, University of Alabama at Birmingham, Birmingham, AL, USA.; ^3^Primary Health Care Centre of Kissamos, Chania, Crete, Greece.; ^4^Department of Hygiene and Epidemiology, Faculty of Medicine, University of Thessaly, Larissa, Greece

## Dear Editor,

Over the last 10 years, public awareness campaigns in organ donation have yielded mixed results, indicating a growing need for culturally-tailored and issue-targeted media campaigns. A 2009 meta-analytic review showed campaigns to be associated with an overall 5% increase in health outcomes, such as registry signing.^1^ A subsequent systematic review indicated that mass media combined with educational interventions could increase organ donor registration and improve knowledge among ethnic minorities in North America and the United Kingdom.^2^ Grass roots and face-to-face efforts, along with targeted health messaging through culturally relevant outreach materials were essential in reducing donor shortages.^3,4^ Long-term effect on minority groups’ knowledge, awareness and perceptions is of particular interest to countries with a growing ethnic or refugee population, undergoing or contemplating reform of their national organ procurement system from informed consent to presumed consent.

The influx of multi-ethnic refugee groups into select South European countries, already under prolonged fiscal constraints, has introduced a new level of complexity for media campaigns. Severe austerity measures have been linked to a rapid decrease in organ donation rates.^5^ Yet, there is ongoing debate whether this is a direct result of the financial crisis or just an exhibit of an already problematic situation.^6^ Organ donation data from Portugal, Italy, and Spain, countries affected by the financial crisis have shown little effect, mainly due to having a well-established national organ procurement system that maintained high donation rates and encouraged contribution from living donors.^5^ On the other hand, data from Greece, undergoing an austerity plan since 2010, have shown a steep decline in the number of solid organ transplantations from deceased donors, from 8-9 donors per million population in 2008 to 4-6 in 2014.^5^ This alarming decrease has also been linked to the 2011 major reform which instituted the presumed consent (opt-out) system in the midst of financial crisis and with limited involvement of stakeholders.^6^ Prior to reform, a survey among primary care patients had showed lack of knowledge and negative attitudes towards registering as informed consent donors.^7^ The survey was based on the hypothesis that inertia would be negated, once opt-out legislation took effect. However, two years later, fewer than 10% of primary care respondents were aware of the newly implemented legislation, while one out of four intended to opt-out.^8^ Moreover, a study of health profession students revealed sub-optimal awareness of the new organ donation system.^9^ Rapidly growing immigration rates from war-torn countries, with refugees vulnerable to being unwittingly subject to presumed consent, added another reason for concern.^6^ Following opposition, the law was modified in 2013 allowing the family of the deceased potential donor to participate in the final decision (soft opt-out). Thus, the organ donation crisis in Greece can be seen as a manifestation of not only the financial crisis, but also of the underlying system inadequacies, underscoring the urgency for carefully planned and targeted information campaigns.

Mass media campaigns aim to affect knowledge, perceptions and attitudes towards a targeted health behavioral change.^10^ To achieve the maximum impact, a campaign should: (*a*) use a theoretical framework to guide the objectives and key messages, (*b*) assess impact on an individual’s intentions to act, and (*c*) deliver behavioral focused messages across to large audiences. A growing body of literature supports use of the theory of planned behavior (TPB) in identifying motivations, designing interventions and addressing racial disparities towards organ donation.^11-13^ According to TPB, interventions targeted at core beliefs are less effective than those targeted at behavior specific beliefs.^14^ In addition, the ‘help-seeking behavior’ has been used to interpret delay or prompt action across an array of health conditions. There is evidence that targeted, well-executed mass media campaigns have small-to-moderate effects not only on health knowledge, beliefs, and attitudes, but also on behaviors.^15^ Hence, increasing an individual’s awareness and knowledge precedes any expected change in behavior. However, the relationship between intention and behavior is still debated, with researchers arguing that persons who intend to take no action regarding a specific behavior, almost always do not carry out the behavior.^16^ At the same time, in an effort to be socially correct or ‘fit,’ some individuals might claim intention to do something but later retreat, when faced with real life difficulties. Thus, the best indicator of a highly effective campaign is achievement of desirable behavioral change, demonstrated through rigorous pre-and-post campaign assessment.^17^ To that extent, a clearly formulated, easy-to-remember campaign slogan can make all the difference. Therefore, widely-used slogans might need to be modified or new ones might need to be tailored to a specific community or population.

All of the above principles should be considered in revamping a public awareness campaign on organ donation. Considering not only the financial constraints, but also the population’s heterogeneity and stage of readiness, is paramount. In the case of Greece, the campaign should reflect the opt-out system’s key differences from the opt-in as ‘talking points.’ Based on evidence that increasing an individual’s awareness and knowledge precedes any expected behavioral change, the main slogan should be *“To be a donor, talk with your family.”* The Ministries of Health, Education, and the Hellenic Transplant Organization, along with regional public health authorities are urged to proceed vigorously, but cautiously. Planning a public awareness campaign should start from the early school years and branch out to older adults, healthcare professionals and other interest groups. Continuous monitoring should ensure that the right messages reach the targeted population at the optimum time. Delivering the message in the most acceptable, appealing, and effective way could make the difference in achieving the desirable behavioral change. Innovative use of information technology, such as social media, mobile phone texting, and websites, as well as emerging digital technologies should be explored in raising awareness.

There are valuable lessons to be learned from the literature, as well as the experience of other pace-setting countries. Rethinking and switching from a “one-size fits all” campaign to community-based targeted health messaging is highly recommended for raising awareness and truly embracing organ procurement reform.

## Ethical issues

Not applicable.

## Competing interests

Authors declare that they have no competing interests.

## Authors’ contributions

EKS conceived the idea and prepared first draft. AM provided theoretical underpinning and finalized manuscript. DA and GR searched the literature and analyzed literature data.

## Authors’ affiliations

^1^Private Family Practice Unit in Heraklion, Heraklion, Crete, Greece. ^2^School of Nursing, University of Alabama at Birmingham, Birmingham, AL, USA. ^3^Primary Health Care Centre of Kissamos, Chania, Crete, Greece. ^4^Department of Hygiene and Epidemiology, Faculty of Medicine, University of Thessaly, Larissa, Greece.
